# Autocrine activity of soluble Flt-1 controls endothelial cell function and angiogenesis

**DOI:** 10.1186/2045-824X-3-15

**Published:** 2011-07-13

**Authors:** Shakil Ahmad, Peter W Hewett, Bahjat Al-Ani, Samir Sissaoui, Takeshi Fujisawa, Melissa J Cudmore, Asif Ahmed

**Affiliations:** 1 University/BHF Centre for Cardiovascular Science, Queen's Medical Research Institute, University of Edinburgh, 47 Little France Crescent, Edinburgh, EH16 4TJ, UK; 2 Department of Reproductive and Vascular Biology, Institute for Biomedical Research, College of Medical and Dental Sciences, University of Birmingham, B15 2TT, UK

## Abstract

**Background:**

The negative feedback system is an important physiological regulatory mechanism controlling angiogenesis. Soluble vascular endothelial growth factor (VEGF) receptor-1 (sFlt-1), acts as a potent endogenous soluble inhibitor of VEGF- and placenta growth factor (PlGF)-mediated biological function and can also form dominant-negative complexes with competent full-length VEGF receptors.

**Methods and results:**

Systemic overexpression of VEGF-A in mice resulted in significantly elevated circulating sFlt-1. In addition, stimulation of human umbilical vein endothelial cells (HUVEC) with VEGF-A, induced a five-fold increase in sFlt-1 mRNA, a time-dependent significant increase in the release of sFlt-1 into the culture medium and activation of the *flt-1 *gene promoter. This response was dependent on VEGF receptor-2 (VEGFR-2) and phosphoinositide-3'-kinase signalling. siRNA-mediated knockdown of sFlt-1 in HUVEC stimulated the activation of endothelial nitric oxide synthase, increased basal and VEGF-induced cell migration and enhanced endothelial tube formation on growth factor reduced Matrigel. In contrast, adenoviral overexpression of sFlt-1 suppressed phosphorylation of VEGFR-2 at tyrosine 951 and ERK-1/-2 MAPK and reduced HUVEC proliferation. Preeclampsia is associated with elevated placental and systemic sFlt-1. Phosphorylation of VEGFR-2 tyrosine 951 was greatly reduced in placenta from preeclamptic patients compared to gestationally-matched normal placenta.

**Conclusion:**

These results show that endothelial sFlt-1 expression is regulated by VEGF and acts as an autocrine regulator of endothelial cell function.

## Background

Vascular endothelial growth factor-A (VEGF-A) is a multifunctional cytokine induced by hypoxic stress [1]. It plays a pivotal role in many aspects of embryonic cardiovascular development, including formation of blood vessels, cardiac morphogenesis, and development of the nervous system [2-6]. Loss and gain of function studies in mice indicate that VEGF-A levels have to be maintained within a narrow range to ensure proper cardiovascular development and embryo survival [7-9]. It has been shown that the effects of VEGF-A can be deleterious if uncontrolled. Over-expression of VEGF in experimental animals increases the leakiness of blood vessels, which may lead to severe edema, loss of limb and death [10,11]. Excess VEGF-A expression in skeletal muscle results in the induction of vascular tumors (hemangiomas) [12-14], whereas loss of VEGF-A activity due to increased production of its natural antagonist, sFlt-1 (soluble VEGF receptor-1/sVEGFR-1), as in preeclampsia, reduces angiogenesis [15]. Thus, homeostasis requires mechanisms to regulate the functional activity of VEGF-A.

Soluble Flt-1 is generated by alternative splicing of the fms-like tyrosine kinase (*flt-1*) gene [16], and binds to all isoforms of VEGF-A and placenta growth factor (PlGF) with high affinity [16,17]. It acts as a potent soluble inhibitor of both VEGF-A and PlGF-mediated biological activities [18] and can also form dominant-negative complexes with competent full-length VEGF receptors [16]. In pregnancies complicated with preeclampsia, sFlt-1 levels are elevated [15,19-21]. Maternal serum levels of sFlt-1 are elevated five weeks prior to the onset of preeclampsia [22], supporting the premise that sFlt-1 is a key factor responsible for the clinical manifestation of this disorder [23]. The demonstration that sFlt-1 is fundamental to the clinical onset of preeclampsia [24] highlights the importance of understanding the intracellular mechanism underlying its regulation and release in endothelial cells. Recently it was shown that autocrine VEGF signaling is required for vascular homeostasis [25]. Here we demonstrate that endothelial sFlt-1 expression is regulated by VEGF and sFlt-1 is an autocrine regulator of endothelial cell function.

## Materials and methods

### Reagents

Recombinant growth factors were purchased from RELIATech (Brauschweig, Germany). Rabbit polyclonal antibodies against phospho- endothelial nitric oxide synthase (eNOS) at serine-1177 (p-eNOS^Ser1177^), phospho-ERK-1/-2 MAPK and phospho-VEGF receptor-2 (VEGFR-2) tyrosine-951 antibodies were purchased from Calbiochem (Nottingham, UK). Small inhibitory RNAs (siRNA) and oligonucleotide primers were purchased from Eurogentec (Southampton, UK). Luciferase reporter assay and cDNA synthesis kits were from Promega (Southampton, UK). All other cell culture reagents and chemicals were obtained from Sigma Aldrich (Poole, UK).

### Placental tissues

Human placental tissue was obtained from normal pregnancies and gestationally-matched pregnancies complicated by preeclampsia. Preeclampsia was defined as blood pressure > 140/90 mm Hg on at least two consecutive measurements and proteinuria of at least 300 mg per 24 hours. Informed consent was obtained from the patients and the study had the approval of the South Birmingham Ethical Committee (Birmingham, UK).

### Cell Culture

Primary human umbilical vein endothelial cells (HUVEC) were isolated and cultured as described [26]. Cells were used at passage two or three for experiments and serum-starved in endothelial cell serum-free medium (Gibco-BRL, UK) supplemented with 0.2% bovine serum albumin for 24 hours prior to stimulation.

### Adenoviral gene transfer

The recombinant, replication-deficient adenoviruses encoding sFlt-1 (Ad-sFlt-1) VEGF (Ad-VEGF) and PTEN (Ad-PTEN) were used as described previously [27-29].

### Quantitative Real-Time PCR

Sample preparation and real-time PCR was performed as described previously [30]. Briefly, mRNA was prepared using TRIzol and DNase-1 digestion/purification on RNAeasy columns (Qiagen), and reverse transcribed with the cDNA Synthesis Kit (Promega). Triplicate cDNA samples and standards were amplified in SensiMix containing SYBR green (Quantace) with primers specific for sFlt-1 [31]. The mean threshold cycle (CT) was normalized to β-actin and expressed relative to control.

### siRNA knock-down of sFlt-1

Two siRNA sequences to the unique 3' sequence of sFlt-1 (sFlt-1 A *sense: *5'-TAACAGUUGUCUCAUAUCAtt-3' and *antisense: *5'-UGAUAUGAGACAACUGUUAtt-3'; sFlt-1 B *sense: *5'-UCUCGGAUCUCCAAAUUUAtt-3' and *antisense *5'-UAAAUUUGGAGAUCCGAGAtt-3') were designed using the Dharmacon *siDESIGN *tool [32]. HUVEC (~ 1 × 10^6 ^cells) were electroporated with ~ 3 μg of sFlt-1, or a universal control siRNA (Dharmacon) using the HUVEC kit II and Amaxa nucleofector (Amaxa GmbH, Cologne, Germany) as described [30].

### Transduction of chimeric VEGF Receptors in HUVEC

A chimeric VEGF/epidermal growth factor (EGF) receptor comprising the intracellular and transmembrane domains of VEGFR-2 fused to the extracellular domain of the human EGF receptor [33]. EGF does not bind to VEGF receptors, therefore, it does not activate the endogenous VEGF receptors. EGDR and its tyrosine-to-phenylalanine mutants (EGDR-Y951F) were generated and cloned into the pMMP retroviral vector, and retrovirus-containing cell supernatant was harvested and used immediately to infect HUVEC [33]. Following 16 hours of incubation, the medium was replaced with fresh growth medium and the HUVEC were used 48 hours after infection.

### Nitric oxide (NO) Release

Total NO in conditioned media was assayed as nitrite, the stable breakdown product of NO, using a Sievers NO chemiluminescence analyzer (Analytix, Sunderland, UK) as described previously [33].

### Tube Formation Assay

The formation of capillary-like structures was examined on growth factor-reduced Matrigel in 24-well plates as described previously [33]. Tube formation was quantified by measuring the total tube length in five random x200 power fields per well using a Nikon phase-contrast inverted microscope with Image ProPlus image analysis software (Media Cybernetics, Silver Spring, USA). Mean total tube length was calculated from three independent experiments performed in duplicate.

### *flt-1 *gene promoter activity assay

A 1.3 Kb fragment of the human *flt-1 *gene corresponding to -1214 to +155 bp relative to the first exon in the pGL2 luciferase vector (Promega) was used to determine *flt-1 *promoter activity [34]. Briefly, porcine aortic endothelial cells (PAEC) were transfected with the *flt-1 *promoter-reporter construct using Exgen 500 (Fermentas, UK) and the cell lysates assayed as described previously [34].

### Western Blotting

Cells lysates were immunoblotted as described previously [33]. Membranes were probed with rabbit polyclonal antibodies against phospho-eNOS-Ser^1177^, anti-ERK-1/-2 or anti-VEGFR-2 phosphotyrosine-951 at 4°C overnight. Proteins were visualised using the ECL detection kit (Amersham-Pharmacia, UK).

### sFlt-1 ELISA

Soluble Flt-1 (sFlt-1) levels in culture supernatants were measured as previously described [30].

### Immunohistochemistry

Formalin-fixed, paraffin-embedded tissues were used for immunohistochemistry as previously described [15].

### Statistical analysis

All data are expressed as mean ± SEM. Statistical comparisons were performed using one-way ANOVA followed by the Student-Newman-Keuls test as appropriate. Statistical significance was set at a value of p < 0.05.

## Results and Discussion

### VEGF-A stimulates sFlt-1 release

**Figure 1:**
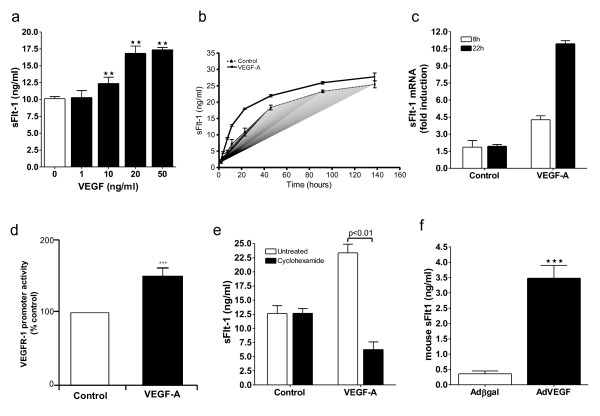
**VEGF stimulates sFlt-1 release from endothelial cells in vitro, and in vivo**. **(a) **VEGF stimulated sFlt-1 release from HUVEC in a concentration-dependent manner. sFlt-1 was measured, in the culture medium, by ELISA, following a 24-hour incubation. **(b) **Time course of VEGF (20 ng/ml) induced sFlt-1 release from HUVEC. **(c) **VEGF induced an increase in sFlt-1 mRNA levels in HUVEC as determined by qPCR and, **(d) ***flt-1 *promoter activity in porcine aortic endothelial cells by luciferase assay. **(e) **Cyclohexamide (10 μg/ml) inhibits VEGF-induced sFlt-1 protein synthesis after 24 hours of treatment. **(f) **Mouse plasma levels of sFlt-1 five days post intravenous injection with an adenovirus encoding VEGF (Ad-VEGF) or control (Ad-βgal). Data are expressed as mean (± SEM) or representative of three or more independent experiments performed in triplicate. **P < 0.01; ***P < 0.001 vs. control.

To evaluate the capacity of VEGF-A to regulate the secretion of its negative regulator, sFlt-1, HUVEC were incubated with VEGF-A and the conditioned media assayed for sFlt-1 by ELISA. VEGF-A stimulated a concentration and time dependent increase in the release of sFlt-1 from HUVEC that reached a maximum at 20 ng/ml and 48 hours, respectively (Figure 1a and 1b). Consistent with these findings, qPCR revealed a greater than five-fold increase in sFlt-1 mRNA after 22 hours of VEGF-A stimulation (Figure 1c). In addition, VEGF-A induced *flt-1 *gene promoter activity in porcine aortic endothelial cells transfected with a *flt-1 *promoter luciferase construct (Figure 1d). Incubation of cells with cycloheximide abrogated the VEGF-A induced response (Figure 1e), which coupled with the fact that there is a negligible release with VEGF-A after two hours of stimulation, indicates that sFlt-1 secretion is due to *de novo *protein synthesis and not release from intracellular vesicles. Adenoviral-mediated overexpression of VEGF-A in mice caused an eight-fold increase in circulating sFlt-1 levels (Figure 1f), demonstrating, *in vivo*, that an increase in VEGF-A results in a concomitant rise in circulating sFlt-1 levels, presumably to compensate for elevated VEGF bioactivity.

### Activation of VEGFR-2, mediates the release of sFlt-1

**Figure 2:**
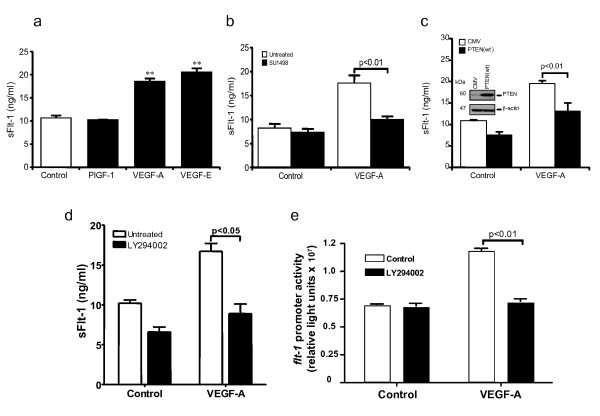
**VEGF-A-induced sFlt-1 release requires VEGFR-2 and the PI3K pathway**. **(a) **sFlt-1 release from HUVEC 24 hours after incubation with PlGF-1 (20 ng/ml), VEGF-A (20 ng/ml) and VEGF-E (20 ng/ml). **(b) **sFlt-1 release from HUVEC after incubation with SU1498 (10 μM), a VEGFR-2 tyrosine kinase inhibitor, prior to addition of VEGF-A (20 ng/ml) for 24 hours. **(c) **sFlt-1 release from HUVEC infected with an adenovirus encoding PTEN (PTEN (wt)) or control adenovirus (CMV) prior to stimulation with VEGF-A (20 ng/ml) for 24 hours. Western blot demonstrating decreased PTEN expression in HUVEC infected with Ad-PTEN(wt) compared with Ad-CMV control (insert). **(d) **sFlt-1 release from HUVEC after incubation with LY294002 (10 μM), a PI3K inhibitor, prior to addition of VEGF-A (20 ng/ml) for 24 hours. **(e) **LY294002 prevented the VEGF-A-induced *flt-1 *promoter activity in porcine aortic endothelial cells, assayed by luciferase reporter assay. Data are expressed as mean (± SEM) or representative of three or more independent experiments performed in triplicate. **P < 0.01 vs. control.

To identify the VEGF receptors involved in the release of sFlt-1, HUVEC were stimulated with either VEGF-A (binds VEGFR-1 and VEGFR-2), or PlGF-1 (binds VEGFR-1) or VEGF-E (binds VEGFR-2). PlGF-1 showed no effect on sFlt-1 release, whereas VEGF-E stimulated similar levels of sFlt-1 release to those induced by VEGF-A (Figure 2a), suggesting that release of sFlt-1 is mediated by VEGFR-2. Preincubation of endothelial cells with SU1498, a VEGFR-2 selective inhibitor, blocked the VEGF-A induced sFlt-1 release (Figure 2b), confirming the importance of this receptor for the response.

### VEGF stimulated sFlt-1 production, is mediated via PI3K

To investigate the role of the PI3K pathway in VEGF-A-induced sFlt-1 release, PI3K activity was inhibited through overexpression of PTEN (Phosphatase and Tensin homolog deleted on chromosome Ten), which dephosphorylates phosphatidylinositol 3,4,5-triphosphate and has been shown to reduce VEGF-mediated signaling and cellular function [28,35]. HUVEC were infected overnight with an adenovirus encoding PTEN (PTEN(wt)) or a control adenovirus (CMV) and stimulated with VEGF-A for 24 hours. Inhibition of PI3K activity by PTEN overexpression led to a significant decrease in sFlt-1 release (Figure 2c). Furthermore, pre-incubation of HUVEC with LY294002, a pharmacological PI3K inhibitor, also attenuated the VEGF mediated release of sFlt-1 (Figure 2d) and of *flt-1 *gene promoter activity (Figure 2e).

### Loss of sFlt-1 promotes angiogenesis

**Figure 3:**
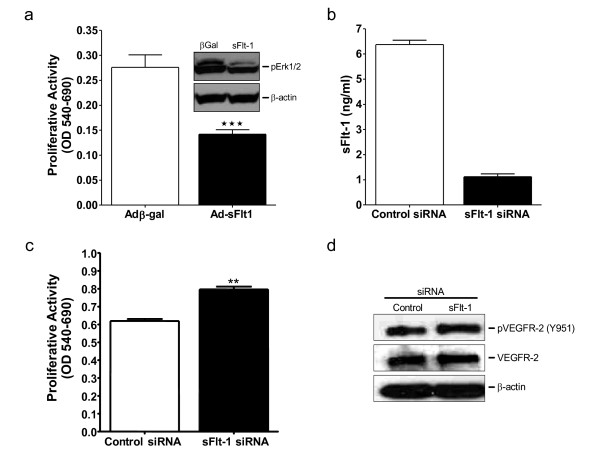
**sFlt-1 inhibits endothelial cell proliferation and VEGF receptor phosphorylation**. **(a) **Cell proliferation after 48 hours of treatment in HUVEC infected with an adenovirus encoding sFlt-1 (Ad-sFlt1) or β-galactosidase control (Ad-βGal). Western blot demonstrating decreased Erk1/2 phosphorylation in HUVEC infected with Ad-sFlt-1 compared with Ad-β-gal control (insert). **(b) **Dramatic reduction of sFlt-1 release from HUVEC 24 hours after transfection with sFlt-1 siRNA. **(c) **Cell proliferation after 48 hours in sFlt-1 siRNA transfected HUVEC. **(d) **Western blot showing VEGF receptor-2 (VEGFR-2) phosphorylation at tyrosine 951 in HUVEC transfected with sFlt-1, or control siRNA. VEGFR-2 and β-actin were used as a loading control. Data are expressed as mean (± SEM) or representative of three or more independent experiments performed in triplicate. **P < 0.01 vs. control.

**Figure 4:**
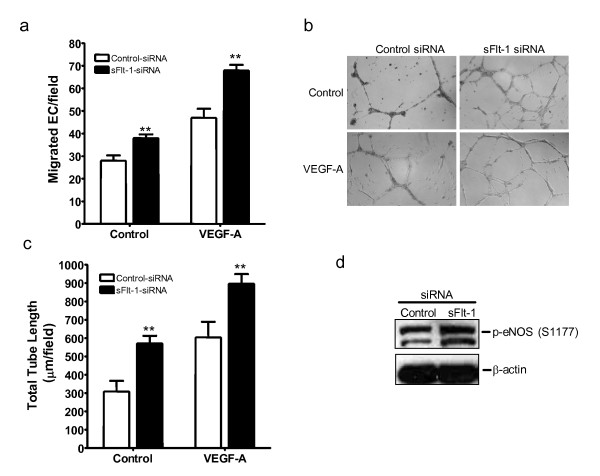
**Loss of sFlt-1 enhances angiogenesis and eNOS phoshorylation**. **(a) **VEGF (20 ng/ml) induced an increase in cell migration of HUVEC transfected with sFlt-1 siRNA compared with control siRNA using a modified Boyden chamber assay. **(b) **VEGF-induced capillary-like tube formation and **(c) **quantification of mean total tube length per field of view after six hours treatment in HUVEC transfected with sFlt-1 siRNA or control. **(d) **Representative Western blot showing eNOS phosphorylation at serine 1177 (p-eNOS (S1177)) in HUVEC transfected with soluble (sFlt-1) or control siRNA. β-actin was used as a loading control. Data are expressed as mean (± SEM) or representative of three or more independent experiments performed in triplicate. **P < 0.01 vs. control.

Adenoviral-mediated overexpression of sFlt-1 in HUVEC inhibited endothelial cell proliferation (Figure 3a) and MAP kinase ERK-1/-2 phosphorylation (Figure 3a insert). Subsequently, to test whether knockdown of sFlt-1 would promote endothelial cell proliferation, HUVEC were transfected with two synthetic siRNA sequences targeted to the unique carboxyl-terminus region of sFlt-1. sFlt-1 siRNA transfection resulted in a substantial reduction in the release of sFlt-1 from HUVEC after 24 hours (Figure 3b). Endothelial cell proliferation was significantly increased (Figure 3c) and interestingly, sFlt-1 knockdown also led to a concomitant increase in VEGFR-2 phosphorylation at tyrosine 951 (Y951) (Figure 3d). In addition, sFlt-1 siRNA increased both basal and VEGF-A-mediated endothelial cell migration (Figure 4a) and tube formation on Matrigel (Figure 4b and 4c). VEGF stimulates eNOS activity and NO release [23,36] to mediate angiogenesis [33,37], thus we predicted that loss of sFlt-1 would increase eNOS phosphorylation in HUVEC. Phosphorylation of eNOS (ser1177) was significantly increased in cells lacking sFlt-1 (Figure 4d). These data provide direct evidence that sFlt-1 is itself a negative regulator of endothelial function. It is likely that sFlt-1 sequesters VEGF and PlGF to maintain a physiological steady state until angiogenesis is required, at which point this system must be overridden.

### Excess sFlt-1 inhibits VEGFR-2 Y951 phosphorylation

**Figure 5:**
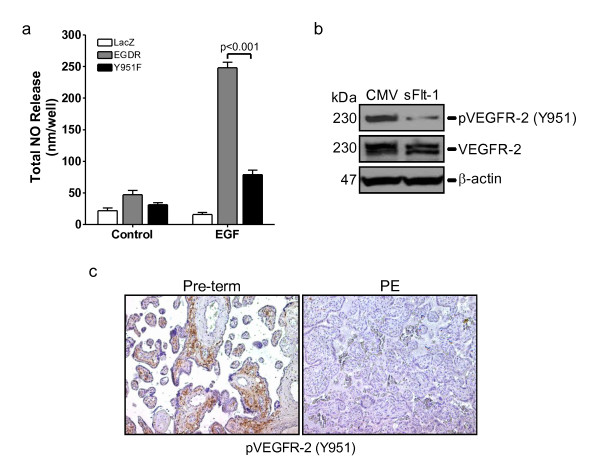
**Excess sFlt-1 inhibits VEGF receptor phosphorylation at tyrosine 951**. **(a) **Nitric oxide (NO) release from HUVEC overexpressing chimeric EGF receptor/VEGFR-2 constructs (EGDR) and EGDR with a mutated Y951 (Y951F), following stimulation with EGF (10 ng/ml) for one hour. NO was measured in cell supernatants using a Sievers chemiluminescent NO analyzer and background subtracted. **(b) **Western blot showing VEGFR-2 and VEGF-2 phosphorylation at tyrosine 951 (p-VEGFR-2 (Y951)) in HUVEC infected with an adenovirus encoding sFlt-1 (sFlt1) or control virus (CMV). β-actin was used as a loading control. **(c) **Representative immunohistochemical staining for p-VEGFR-2 (Y951) in gestationally-matched normal (pre-term) and preeclamptic (PE) placenta. Data are expressed as mean (± SEM) or representative of three or more independent experiments performed in triplicate.

Activation of VEGFR-2 leads to an increase in eNOS expression and activation,[38] which is essential for neovascularisation [37]. A recent study showed that mutation of VEGFR-2 Y951 to phenylalanine caused a significant reduction in VEGF-induced angiogenesis [33]. As preeclampsia is associated with elevated placental [15] and circulating [22] sFlt-1 and placental sFlt-1 inhibits angiogenesis [15], we speculated that elevated free sFlt-1 would lead to a reduction of VEGFR-2 phosphorylation in preeclamptic placenta. Using the EGFR-chimeric receptor system we show that mutation of Y951 to phenylalanine resulted in over 50% reduction in NO release (Figure 5a) and overexpression of sFlt-1 in endothelial cells abrogated phosphorylation of VEGFR-2 Y951 (Figure 5b). To assess whether VEGFR-2 phosphorylation was reduced in preeclamptic placenta, that express elevated sFlt-1, we undertook immunohistochemical analysis for phospho-VEGFR-2 Y951. Overall, phosphorylation of VEGFR-2 Y951 was greatly reduced in the preeclamptic placenta compared to gestationally-matched, normal placenta (Figure 5c). Together, these findings indicate that increased levels of sFlt-1 have a negative effect on VEGFR-2 tyrosine phosphorylation, which in turn would lead to concomitant inhibition of downstream function and signaling and compromise maternal vascular homeostasis and placental angiogenesis.

## Conclusions

Endothelial cell sFlt-1 expression is regulated by VEGF and sFlt-1 is an autocrine regulator of endothelial cell function.

## Abbreviations

**EGF**: epidermal growth factor; **eNOS**: endothelial nitric oxide synthase; **ERK-1/-2**: extracellular signal regulated kinase -1/2; **HUVEC**: human umbilical vein endothelial cells; **MAPK**: mitogen activated kinase; **NO**: nitric oxide; **PI3K**: phosphoinositide-3'-kinase; **PlGF**: placenta growth factor; **PTEN**: Phosphatase and Tensin homolog deleted on chromosome Ten; **sFlt-1**: soluble vascular endothelial growth factor receptor-1; **VEGF-A**: vascular endothelial growth factor-A; **VEGFR-1**: vascular endothelial growth factor receptor-1; **VEGFR-2**: vascular endothelial growth factor receptor-2.

## Competing interests

The authors declare that they have no competing interests.

## Authors' contributions

SA, MJC, PWH, BA, SS and TF performed the experiments and analysed the data SA PWH and MJC contributed to the writing of the manuscript. AA designed experiments and wrote the manuscript. All authors read and approved the final manuscript.
